# Corrosion Behavior of 10 ppi TAD_3D_/5A05Al Composite in a Chloride Environment

**DOI:** 10.3390/ma17061280

**Published:** 2024-03-10

**Authors:** Zishen Li, Shengpu Wang, Yuxin Chen, Gaofeng Fu, Lan Jiang

**Affiliations:** 1 Key Laboratory for Ecological Metallurgy of Multimetallic Mineral, Ministry of Education, Northeastern University, Shenyang 110819, China; 2 School of Metallurgy, Northeastern University, Shenyang 110819, China; 3 China Minmetals Nonferrous Metals Co., Ltd., Beijing 100044, China

**Keywords:** aluminum dross, TAD_3D_/5A05Al, composite, neutral salt spray, corrosion behavior, electrochemistry

## Abstract

This study utilizes desalted and denitrated treated aluminum dross (TAD) as a raw material, along with kaolin and 10 ppi (pores per inch) polyurethane foam as a template. The slurry is converted into an aluminum dross green body with a three-dimensional network structure using the impregnation method. A three-dimensional network aluminum dross ceramic framework (TAD_3D_) is created at a sintering temperature of 1350 °C. The liquid 5A05 aluminum alloy at a temperature of 950 °C infiltrates into the voids of TAD_3D_ through pressureless infiltration, resulting in TAD_3D_/5A05Al composite material with an interpenetrating phase composite (IPC) structure. The corrosion behavior of TAD_3D_/5A05 composite material in sodium chloride solution was examined using the salt spray test (NSS) method. The study shows that the pores of the TAD3D framework, produced by sintering aluminum dross as raw material, are approximately 10 ppi. The bonding between TAD3D and 5A05Al interfaces is dense, with strong interfacial adhesion. The NSS corrosion time ranged from 24 h to 360 h, during which the composite material underwent pitting corrosion, crevice corrosion and self-healing processes. Results from Potentiodynamic Polarization (PDP) and Electrochemical Impedance Spectroscopy (EIS) indicate that, as corrosion progresses, the *E*_corr_ of TAD_3D_/5A05Al decreases from −0.718 V to −0.786 V, and *I*_corr_ decreases from 0.398 μA·cm^−2^ to 0.141 μA·cm^−2^. A dense oxide film forms on the surface of the composite material, increasing the anodic Tafel slope and decreasing the cathodic Tafel slope, thus slowing down the rates of cathodic and anodic reactions. Factors such as lower interface corrosion resistance or a relatively weak passivation film at the interface do not significantly diminish the corrosion resistance of TAD_3D_ and 5A05Al. The corrosion resistance of the composite material initially decreases and then increases.

## 1. Introduction

The 5A05Al aluminum alloy belongs to the Al-Mg series, exhibiting high strength, excellent corrosion resistance, formability, and weldability. It finds extensive applications in transportation and mechanical engineering fields [1]. To enhance the wear resistance of 5A05 aluminum alloy, researchers have developed ceramic particles, ceramic whiskers, and ceramic frameworks to reinforce 5A05Al composite materials [2]. Such composite materials boast advantages including high temperature strength, elevated wear resistance, and high hardness [3]. Aluminum oxide (Al_2_O_3_) possesses excellent properties such as a high modulus, wear resistance, strength, and low density. Introducing aluminum oxide particles or whiskers into 5A05 aluminum alloy can significantly enhance its high-temperature and wear-resistant performance [4]. However, uneven dispersion of Al_2_O_3_ particles and whiskers in the composite material leads to a significant reduction in the material’s mechanical properties, wear resistance, and corrosion resistance [5]. Al_2_O_3_ is shaped into a three-dimensional network ceramic framework (Al_2_O_33D_). Subsequently, an aluminum alloy solution is infiltrated into the pores of the ceramic framework to prepare a three-dimensional network Al_2_O_3_ ceramic framework-reinforced aluminum-based composite material, Al_2_O_33D_/Al. In this material, Al_2_O_33D_ and Al interpenetrate in three-dimensional space, exhibiting isotropy and a unique structure [6]. Such materials are referred to as interpenetration phase composites (IPC) [7]. When external forces are applied to the Al_2_O_33D_/Al composite material with an IPC structure, the interaction forces between Al_2_O_33D_ ceramic and 5A05Al metal are effectively dispersed, absorbed, and dissipated. Consequently, the composite material can simultaneously leverage the high hardness, wear resistance, and thermal resistance of Al_2_O_3_ ceramic in three-dimensional space, as well as the high toughness and excellent thermal conductivity of 5A05 aluminum alloy. Research indicates that Al_2_O_33D_ reinforcement significantly enhances the high-temperature resistance and wear resistance of the friction surface of aluminum-based composite materials [8,9]. Additionally, it maintains the low density, high thermal conductivity, and high specific strength of the composite material. Therefore, Al_2_O_33D_/5A05Al composite materials, such as for brake discs, exhibit tremendous potential [10]. Recently, researchers have utilized industrial solid waste aluminum dross as raw material for alumina-based ceramic material production [11]. Aluminum dross is an industrial residue generated during aluminum smelting, typically with an Al_2_O_3_ content exceeding 70%. If aluminum dross can be used to produce porous aluminum dross ceramic materials (TAD_3D_) instead of Al_2_O_33D_, it would bring significant benefits to the high-value utilization of solid waste and cost reduction in ceramic-reinforced aluminum-based composite materials [12].

Typically, methods for preparing IPC include pressureless infiltration, squeeze casting, and vacuum pressure infiltration, among others [13]. Squeeze casting and vacuum pressure infiltration processes are more complex, while the pressureless infiltration method for preparing IPC is relatively simple and enables low-cost industrial applications [14]. However, if the pressureless infiltration method is used to prepare TAD_3D_/5A05Al, and the wetting property between TAD_3D_ and 5A05Al is poor, numerous defects may occur at the interface of TAD_3D_ and 5A05Al, significantly reducing the overall performance of the composite material [15]. Moreover, when TAD_3D_/5A05Al IPC is used as a friction material, such as brake discs, it often operates in environments with water mist, salt spray, etc., for extended periods. Interface defects typically reduce the material’s corrosion resistance, leading to issues like pitting corrosion, crevice corrosion, and intergranular corrosion (IGC) [16]. TAD_3D_/5A05Al exhibits diverse phases, making its corrosion behavior intricate. Studies indicate that moderate levels of Mn, Mg, and Zn are advantageous for improving the corrosion resistance of aluminum alloys. Conversely, Fe, Cu, and Ni tend to decrease the corrosion resistance, while the impact of Si and Ti on aluminum alloy corrosion resistance varies depending on the alloy type and environment [17,18,19].

The presence of Mg significantly enhances the wettability of aluminum oxide ceramic with aluminum alloy solutions [20]. As 5A05 contains Mg, it markedly improves the wetting property between TAD_3D_ and 5A05Al, enabling the possibility of preparing TAD_3D_/5A05Al using the pressureless infiltration method. However, there is limited literature on the pressureless infiltration method for preparing TAD_3D_/5A05Al IPC, making the study of the pressureless infiltration preparation of TAD_3D_/5A05Al and its corrosion-resistance behavior of great significance. This study employs aluminum dross as the aluminum source, kaolin as the silicon source, and utilizes a 10 ppi polyurethane foam as the template to prepare TAD_3D_ using the sacrificial template method. The 5A05Al alloy solution is pressureless infiltrated into TAD_3D_ to produce TAD_3D_/5A05Al. The microstructure of TAD_3D_/5A05Al and its neutral salt spray (NSS) corrosion performance at different time intervals are investigated using an electrochemical workstation, optical microscope, scanning electron microscope (SEM). The composite material prepared by pressureless infiltration exhibits minimal casting defects, and the interface between TAD_3D_ and 5A05Al is tightly bonded, ensuring excellent corrosion resistance. This approach realizes the high-value utilization of aluminum dross solid waste, providing a crucial theoretical foundation for the application of aluminum dross ceramic-reinforced aluminum-based composite materials in the fields of long-lasting wear resistance and corrosion resistance.

## 2. Materials and Methods

### 2.1. Experimental Materials

This study utilizes treated aluminum dross as the main raw material. The TAD_3D_ ceramic scaffold is prepared using the polyurethane foam template method, and the composite material is fabricated by pressureless casting of TAD_3D_ with 5A05Al. The composite material samples undergo corrosion through the NSS method. This study explores the corrosion behavior of the composite material in a chloride environment by observing surface morphology, analyzing surface composition, and conducting electrochemical measurements on samples at different corrosion times.

The treated aluminum dross powder (Inner Mongolia Hengsheng Environmental Protection Technology Co., Ltd., Tongliao, China) and kaolin powder (Lingshou County Huayao Mineral Products Processing Factory, Shijiazhuang, China) serve as the primary raw materials. Their compositions are shown in Table 1. Dolapix CE-64 (Zschimmer-Schwarz (China) Ltd., Foshan, China) serves as a dispersant, Polyvinyl alcohol (PVA, Changchun Chemical (Jiangsu) Co., Ltd., Changshu, China) functions as a binder, and carboxymethyl cellulose (CMC, Shanghai Shenguang Edible Chemicals Co., Ltd., Shanghai, China) acts as a thickener. The treated aluminum dross, kaolin, Dolapix CE-64, CMC, and PVA were mixed in weight percentages of 80 wt.%, 17 wt.%, 2 wt.%, 0.5 wt.%, and 0.5 wt.%, respectively. The mixture was added to 100 mL distilled water, poured into a nylon jar equipped with 10 mm diameter ZrO_2_ balls, ball-milled for 12 h, resulting in a slurry with a solid content of approximately 70%. Adjust the pH of the slurry to 9.1–9.8 using ammonia water. Immerse a 10 ppi (10 pores per inch) polyurethane open-cell sponge template with a diameter Φ = 100 mm and thickness h = 30 mm into the slurry, and remove excess slurry using a double-roll extrusion. Compressed air is blown over the polyurethane sponge covering the slurry to eliminate remaining closed pores. After drying in a microwave for 15–20 min, a porous network body with good strength is obtained. The polyurethane sponge is visibly coated with a light gray 0.8–1 mm thick layer of aluminum dross. The body is placed in a high-temperature box resistance furnace (KSL1700X, Hefei Kejing Material Technology Co., Ltd., Hefei, China), heated from 25 °C to 1350 °C at a rate of 5 °C/min, and sintered at 1350 °C for 3 h to obtain TAD_3D_.

The TAD_3D_/5A05Al composite material was prepared using the pressureless casting method [21]. The 5A05Al alloy (Guangxi Nannan Aluminum Processing Co., Ltd., Nanning, China) with its chemical composition is shown in Table 2. The 5A05Al aluminum alloy block was placed in a corundum crucible, and the crucible along with the TAD_3D_ block was positioned in a crucible resistance furnace (SG-2, Henan Eng Furnace Machinery Equipment Co., Ltd., Zhengzhou, China), with the temperature raised to 950 °C. The residue from the surface of the 5A05 Al in the crucible was removed, and the aluminum melt was stirred uniformly. TAD_3D_ was placed into the aluminum melt, the crucible was tightly pressed with refractory materials and maintained at 950 °C for 30 min. The 5A05Al melt fully infiltrated into TAD_3D_, and after cooling, TAD_3D_/5A05Al was obtained. TAD_3D_/5A05Al specimens with dimensions of 10 × 10 × 6 mm^3^ were cut using a metallographic cutting machine (Laizhou Weiyi Machinery Manufacturing Co., Ltd., Yantai, China) for the study of the microstructure, electrochemical properties, and NSS corrosion performance of the composite material.

### 2.2. Neutral Salt Spray Corrosion

The NSS was conducted using an automated neutral salt spray chamber (ZK-60K, Dongguan Zhenke Testing Equipment Co., Ltd., Dongguan, China). The corrosive solution consisted of 5 wt.% with a pH range of 6.5 to 7.2 in a neutral NaCl solution. Experimental durations were set at 24 h, 72 h, 144 h, 240 h, and 360 h, with a test temperature of (35 ± 1) °C.

### 2.3. Surface Morphology of the Corroded Composite

After NSS corrosion, the corrosion microstructures on the sample surface were observed using an electron microscope equipped with an energy-dispersive X-ray spectrometer (EDS) (HITACHI S4800, Tokyo, Japan), and the composition of the corrosion products was analyzed.

### 2.4. Electrochemical Measurement

The electrochemical properties of TAD_3D_/5A05Al samples corroded under NSS for various durations were investigated using an electrochemical workstation (CHI 790E, Shanghai Chenhua Instrument Co., Ltd., Shanghai, China). Electrochemical testing comprised polarization curves (PDP) and electrochemical impedance spectroscopy (EIS), conducted using a three-electrode system. A standard calomel electrode (saturated potassium chloride solution) served as the reference electrode, with a platinum foil employed as the auxiliary electrode [19]. The TAD_3D_/5A05Al was connected to copper wire, sealed with epoxy resin, and used as the working electrode. The area of the test sample was 1 cm^2^. At room temperature, the sample was immersed in a 3.5% NaCl solution for 30 min. After stabilizing the open-circuit potential (OCP), polarization curves (PDP) and electrochemical impedance spectra (EIS) were measured. A scan rate of 0.25 mV/s was used for the cathodic-to-anodic scan, covering the range of OCP ± 600 mV for PDP measurements. During EIS measurements, a frequency range of 10^−2^–10^6^ Hz was employed, with a sinusoidal perturbation signal of 10 mV added. The data were fitted using ZView software (Ver. 2.70) after the tests.

## 3. Results and Discussion

### 3.1. Microstructure of TAD_3D_/5A05Al

**Figure 1:**
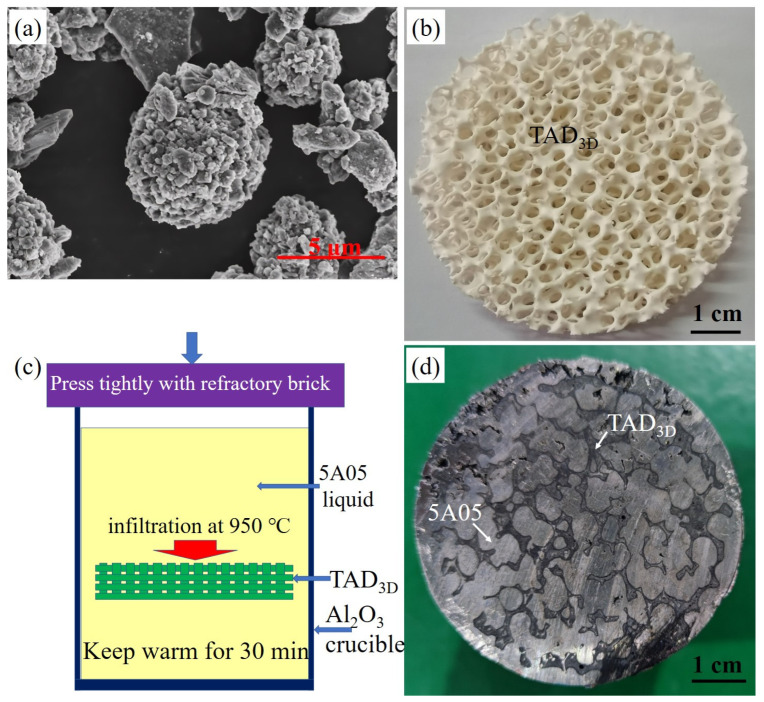
The raw materials, process, and samples for preparing TAD_3D_/5A05Al composite materials: (**a**) SEM of treated aluminum dross; (**b**) TAD_3D_; (**c**) schematic of the pressureless infiltration process; (**d**) TAD_3D_/5A05Al.

Figure 1 depicts the raw materials, process schematic, and sample photos for the preparation of TAD_3D_/5A05Al composite material. In Figure 1a, an SEM image of the treated aluminum dross is shown, revealing its spherical shape. The sintered TAD_3D_ framework is shown in Figure 1b, with a shrinkage rate of approximately 18% after sintering the green body. The framework appears yellowish-white, indicating successful sintering into ceramics; it exhibits a measured compressive strength of around 2–5 MPa, and a pore size of approximately 10 ppi. Figure 1c illustrates the schematic of the pressureless infiltration process, where the TAD_3D_ framework guides the molten 5A05Al alloy, effectively expelling fine air bubbles between the framework and the 5A05Al solution, preventing defects in the internal structure of the composite material and reducing internal stresses during the preparation process. The samples of TAD_3D_/5A05Al composite material exhibit a reflective silver-white luster and produce a solid tapping sound when cut to obtain test specimens, as shown in Figure 1d. On the smooth surface, gray streaks represent the framework, while the silver-white metallic luster corresponds to the 5A05Al alloy. The TAD_3D_ framework has good affinity and wetting properties with the 5A05Al alloy, resulting in a dense interface and strong interfacial bonding. The samples of TAD_3D_/5A05Al composite material exhibit a reflective silver-white luster and produce a solid tapping sound when cut to obtain test specimens, as shown in Figure 1d. On the smooth surface, gray streaks represent the framework, while the silver-white metallic luster corresponds to the 5A05Al alloy. The TAD_3D_ framework exhibits excellent wetting properties with the 5A05Al alloy solution, and a dense bond is formed at the interface between TAD_3D_ and 5A05Al, resulting in high bonding strength.

**Figure 2:**
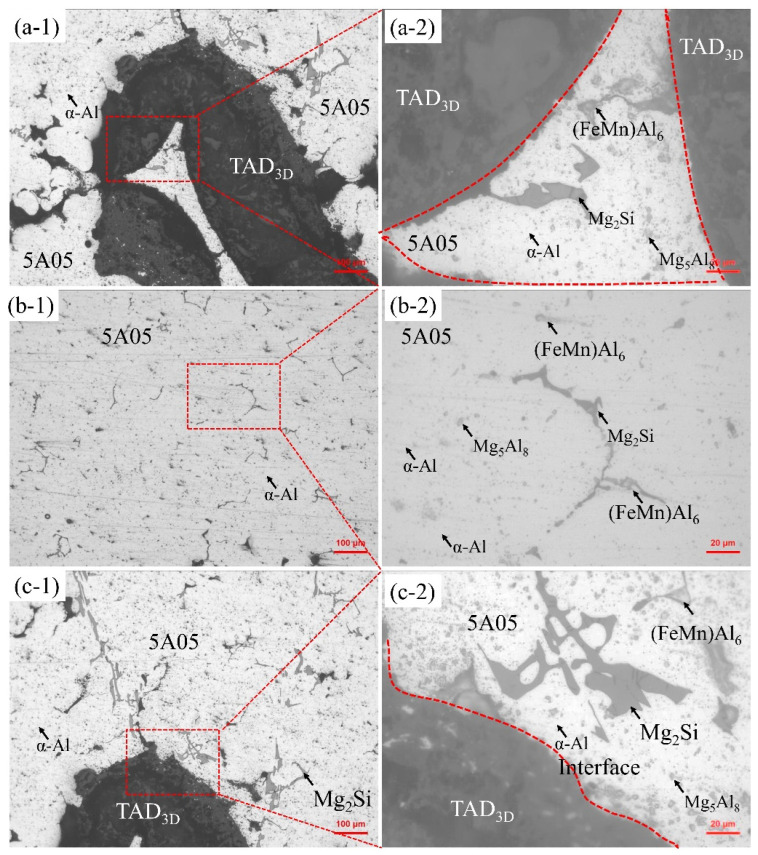
Optical micrograph (OM) image of TAD_3D_/5A05Al: (**a-1**,**a-2**) TAD_3D_; (**b-1**,**b-2**) 5A05Al; (**c-1**,**c-2**) composite interface.

Figure 2 illustrates the microstructure of the non-corroded TAD_3D_/5A05Al composite material. In Figure 2a-1, the microstructure of TAD_3D_ in the composite material is shown. Figure 2a-2 is an enlarged view of Figure 2a-1, revealing that the pores in the ceramic phase are completely filled by the 5A05 aluminum alloy. TAD_3D_, manufactured by the polyurethane foam template method, contains triangular voids left by the combustion of polyurethane foam. In this study, the pressureless infiltration method was used to completely fill the triangular voids in TAD_3D_ with the 5A05Al solution, achieving a dense IPC structure between the 5A05Al alloy and TAD_3D_, significantly enhancing the mechanical and corrosion resistance properties of TAD_3D_/5A05Al. In Figure 2a-2, the crystal boundaries and intra-crystalline regions of 5A05Al within the triangular voids are clearly visible, showing numerous second-phase particles, including the β-phase Mg_5_Al_8_, Mg_2_Si, and (FeMn)Al_6_. In 5A05 aluminum alloy, Si and Mg react to form Mg_2_Si according to Equation (1), while Al and Mg react to produce the β-phase Mg_5_Al_8_ according to Equation (2). The coarse needle-like or plate-like AlMnMgSi phase, generated by the elements Al, Mn, Mg, and Si, represents a non-equilibrium solidification microstructure [22].

Si + 2Mg → Mg_2_Si (1)

8Al+ 5Mg → Mg_5_Al_8_ (2)

Figure 2b-1,b-2 depicts the microstructure of 5A05Al, revealing that the α-Al grains of 5A05Al contain relatively fewer second-phase particles such as Mg_2_Si, β-phase Mg_5_Al_8_, and (FeMn)Al_6_. The Mg_2_Si grains are small, approximately 3 μm, while Mg_5_Al_8_ is around 3 μm, Mg_2_Si is approximately 30 μm, and (FeMn)Al_6_ is about 15 μm.

Figure 2c-1,c-2 shows the microstructure of the interface between TAD_3D_ and 5A05Al alloy. The interface between the 5A05Al alloy and TAD_3D_ is clear, with a tight bond. At the interface, the quantity of second-phase particles, including Mg2Si, Mg_5_Al_8_, and (FeMn)Al_6_, increases, and the Mg_2_Si precipitates become coarser, forming a Chinese character pattern. Due to the different solubility of alloy elements in solid and liquid phases, elements such as Si, Mg, Fe, Mn in the 5A05 aluminum alloy tend to segregate into the liquid around the dendritic arms. Segregation alters the local thermodynamics of the alloy, providing a driving force for the formation of a second phase in the interdendritic region. Therefore, the second-phase particles, including Mg_2_Si, Mg_5_Al_8_, and (FeMn)Al_6_, increase in the interface between the alloy and the TAD_3D_.

By complementing the TAD_3D_ ceramic network and 5A05A with their own advantages, the TAD_3D_/5A05Al composite can provide the desirable mechanical properties including high specific stiffness, high plastic flow strength, creep resistance, good oxidation, and corrosion resistance.

**Figure 3:**
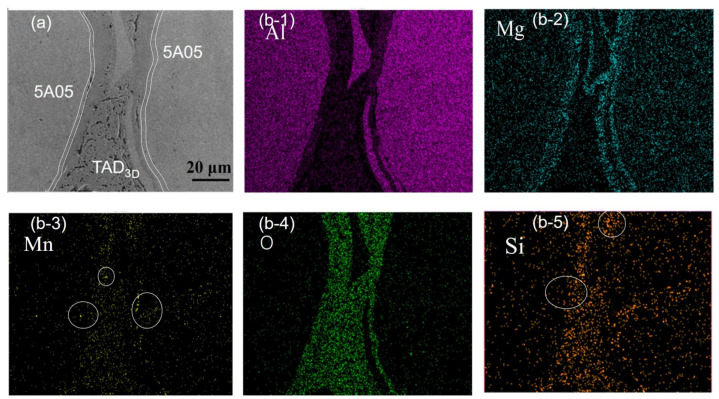
SEM and EDS of TAD_3D_/5A05Al: (**a**) SEM of composite interface; (**b-1**) Al; (**b-2**) Mg; (**b-3**) Mn; (**b-4**) O; (**b-5**) Si.

**Figure 4:**
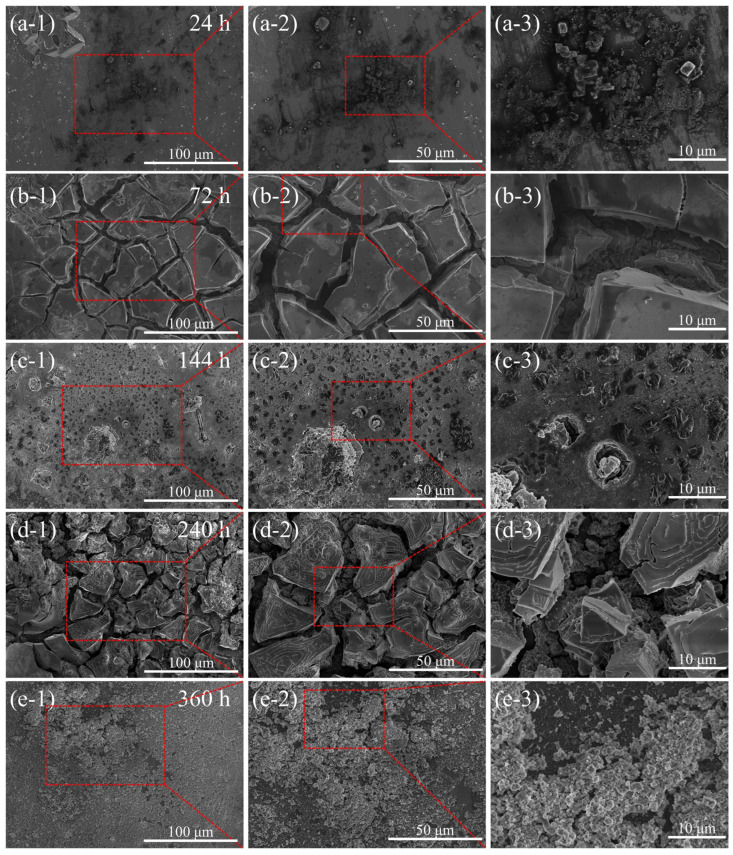
SEM images of TAD3D/5A05Al with corrosion products after NSS corrosion for different durations: (**a-1**–**a-3**) 24 h; (**b-1**–**b-3**) 72 h; (**c-1**–**c-3**) 144 h; (**d-1**–**d-3**) 240 h; (**e-1**–**e-3**) 360 h.

**Figure 5:**
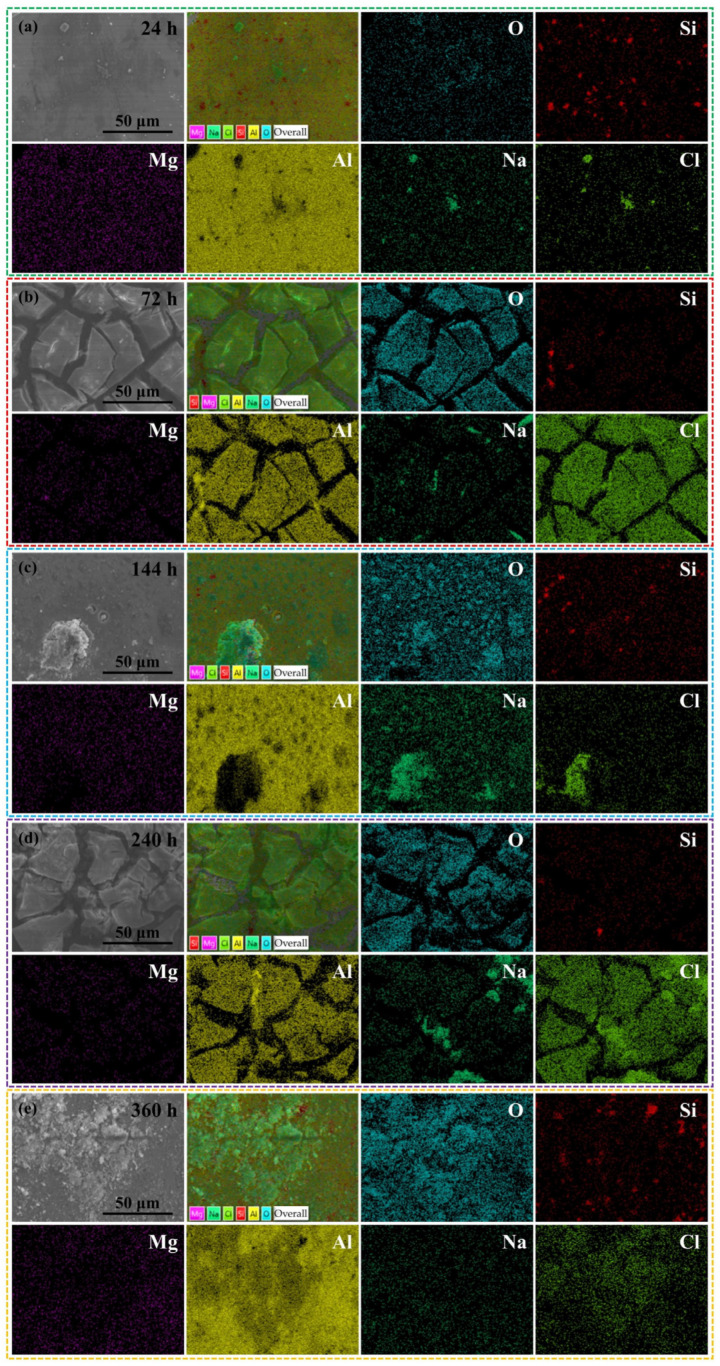
EDS of TAD3D/5A05Al with corrosion products after NSS corrosion for different durations: (**a**) 24 h; (**b**) 72 h; (**c**) 144 h; (**d**) 240 h; (**e**) 360 h.

Figure 3 depicts SEM and EDS surface scan images of the non-corroded composite material. The dense interface between the 5A05Al alloy and TAD_3D_ in the composite material is clearly visible. An interface layer, corroded by the etching solution, can be observed between TAD_3D_ and the 5A05Al matrix, as indicated by the white dashed line in Figure 3a, with a thickness of approximately 4 μm. The EDS analysis of Figure 3b-1–b-5 indicates that the 5A05Al alloy is mainly composed of Al, Mn, Mg, and Si elements. Mg diffuses from the 5A05Al matrix to TAD_3D_ and accumulates in TAD_3D_. The distribution of O elements in the 5A05Al matrix is uniform, while in TAD_3D_, it exhibits enrichment. Mg and Si elements form granular Mg_2_Si phases and excess Si particles. Si elements in the 5A05Al matrix precipitate in small amounts, exhibiting a punctate aggregation. Mg and Al elements form Mg_5_Al_8_ phases, and Fe, Mn, Al, and other elements form (FeMn)Al_6_ phases in TAD_3D_. TAD_3D_/5A05Al exhibits a slender network distribution of precipitated Mg_2_Si [23].

### 3.2. Microstructure of the Composite Material with NSS Corrosion Products

Figure 4 presents SEM images of the corroded surface of TAD_3D_/5A05 composite materials after NSS corrosion for 24 h, 72 h, 144 h, 240 h, and 360 h, respectively.

Figure 4a-1–a-3 reveal that after 24 h of NSS corrosion, there are relatively few pitting corrosion pits on the metal surface at the interface between TAD_3D_ and 5A05Al. Pitting corrosion in the 5A05Al matrix generates, expands, and connects with each other, forming larger pitting corrosion pits. The corrosion products of 5A05 aluminum alloy increase, forming sheet-like accumulations on the corroded surface. Figure 4b-1–b-3 illustrates that after 72 h of NSS corrosion, corrosion products are generated on the surface of the 5A05Al matrix, accompanied by the formation of a passive film. Cracks are observed on the passive film, exhibiting a relatively uniform pattern with a length of approximately 50 μm and a width of about 5 μm. The cracks create an oxygen-deficient zone, where Cl^−^ accumulates in the solution, leading to a different metal surface state inside the cracks compared to the exterior. The solution inside the cracks transitions from neutral to acidic, resulting in crevice corrosion in these areas. Figure 4c-1–c-3 illustrates the condition of pitting corrosion on the 5A05Al matrix after 144 h of NSS corrosion. The diameters of these pitting pits have expanded laterally, increasing from 1 μm to 5 μm, with simultaneous growth in depth and area. The increased adsorption of Cl^−^ on the 5A05Al matrix results in the formation of more pitting pits on the surface. These pits expand, leading to the merging of multiple small pitting pits into larger ones. Some large pitting pits retain a small amount of residual corrosion products. Figure 4d-1–d-3 show that after 240 h of NSS corrosion, a secondary reaction occurs on the 5A05Al matrix, leading to the formation of an aluminum oxide film [24], which enhances the passivation performance of the metal, resulting in improved corrosion resistance. Destructive separation behavior due to crevice corrosion is prevented. Figure 4e-1–e-3 reveal that after 360 h of NSS corrosion, the larger pitting corrosion sites on the corroded sample surface are covered by corrosion products, leading to self-healing of the corroded surface and a significant enhancement in corrosion resistance [25]. We preliminarily believe that the reason for self-healing is that TAD_3D_ sintered from aluminum dross has strong hydration reaction ability. TAD_3D_ reacts with water to form aluminum hydroxide gel with high strength. At the same time, MgAl_2_O_4_, Al(OH)_3_, and Al_2_O_3_ are filled in the gaps between TAD_3D_ and 5A05, as well as in the pitting pits on the surface, and together with the TAD_3D_ skeleton embedded in 5A05Al, form a dense and sturdy passivation film. This results in a self-healing phenomenon at the interface of composite materials, enhancing their corrosion resistance.

Figure 5 presents the EDS analysis results of TAD_3D_/5A05 after NSS corrosion for 24 h, 72 h, 144 h, 240 h, and 360 h. EDS analysis results after 24 h of NSS corrosion presented in Figure 5a indicate that the main components of the corrosion products are O, Si, Mg, Al, Na, and Cl, suggesting that the corrosion product is Al(OH)_3_ [23]. The uniformly distributed O element on the 5A05Al matrix reacts with H and Al according to Equation (3) to produce Al(OH)_3_.

Al + 3O + 3H → Al(OH)_3_ (3)

Figure 5b presents the EDS analysis results after 72 h of NSS corrosion, indicating that the corrosion products are Al(OH)_3_ or corrosion products containing Mg. The morphology of the corrosion products is crack-like, and this feature is formed by the continuous generation of corrosion products in the active corrosion zone, gradually spreading and diffusing towards the surrounding area [26]. EDS analysis of the corrosion products indicates that the main elemental components in the corrosion products are Al, O, Cl, Mg, and Si. Due to the presence of interface reaction products and defects at the interface of TAD_3D_ and the 5A05Al matrix, it becomes an active site. The Al matrix, as the anodic region, undergoes dissolution reactions first, forming Al^3+^ and releasing electrons, while the cathodic region undergoes oxygen absorption reactions forming OH^−^. The electrochemical reactions are shown in Equations (4) and (5).

Al → Al^3+^ + 3e^−^ (4)

O_2_ + 2H_2_O + 4e^−^ → 4OH^−^ (5)

Some of the Al^3+^ in the solution reacts with OH^−^ to generate Al(OH)_3_, and it can further react to form Al_2_O_3_ through Equation (6).

2Al(OH)_3_ → Al_2_O_3_ + 3H_2_O (6)

The formation of Al(OH)_3_ and Al_2_O_3_ through reactions has elevated the corrosion potential of the composite material, reducing the corrosion rate.

Figure 5c shows the EDS analysis results after 144 h, indicating the adsorption of Na^+^ and Cl^−^ ions at the interface, and some pitting corrosion extending to the interface between TAD_3D_ and 5A05Al. The pitting corrosion is significantly deepened compared to NSS corrosion at 72 h, and the pits on the metal matrix develop into larger cracks, causing damage to the interface. At this point, corrosion intensifies [27].

Figure 5d shows the NSS corrosion for 240 h. EDS analysis of the corrosion products inside the pitting reveals that the predominant components are O and a small amount of Al, with overlapping states of the corrosion product Al(OH)_3_. Compared to 144 h, the number of cracks on the metal matrix surface decreases, and the corrosion behavior at the interface is mainly characterized by the healing of small cracks on the passivation film [28]. With the extension of NSS exposure, the oxide film on the metal surface gradually thins. Cl^−^ reacts directly with 5A05Al through tiny pores in the oxide film, forming a gray-white corrosion product AlCl_3_. Therefore, it can be inferred that the corrosion products mainly consist of Al(OH)_3_, Al_2_O_3_, and AlCl_3_.

Figure 5e shows NSS corrosion after 360 h, where some pits on the surface of the 5A05Al matrix are covered by corrosion products, improving the corrosion resistance of the material. EDS analysis of the corrosion products at the interface indicates the presence of O, Al, and Mg, suggesting the existence of MgAl_2_O_4_ spinel in the corrosion products. The corrosion products MgAl_2_O_4_, Al(OH)_3_, Al_2_O_3_, and AlCl_3_ adsorb at the interface, generating an oxide film along with the TAD_3D_ ceramic phase. This oxide film serves as an insulating barrier, restricting the inward diffusion of corrosive substances (such as ions or moisture) into the Al alloy matrix. It demonstrates a synergistic effect resulting from the combination of TAD_3D_ and 5A05Al in IPC composite materials, forming a stronger and more resistant passive layer that enhances the corrosion resistance of the composite material.

### 3.3. Microstructure of the Composite Material after Removal of NSS Corrosion Products

**Figure 6:**
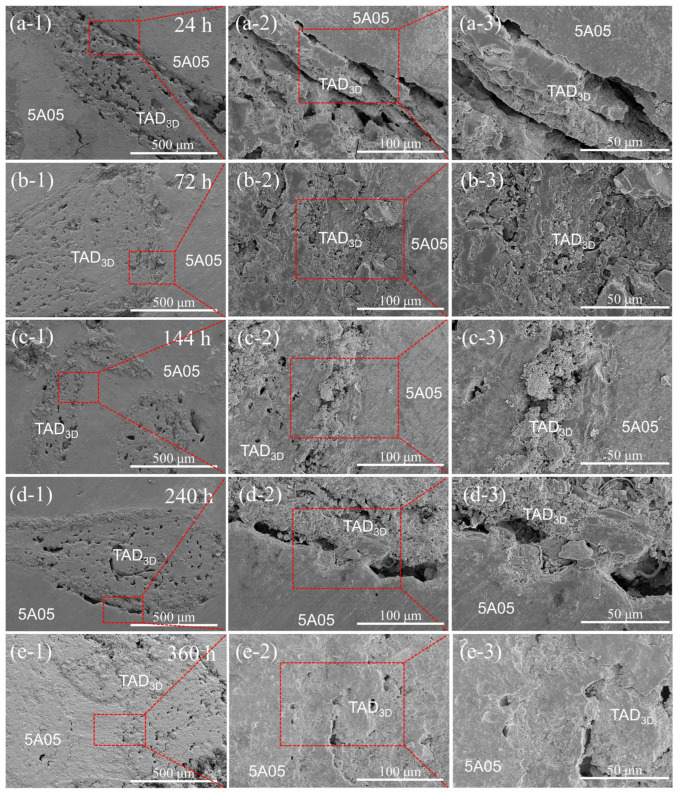
SEM images of TAD_3D_/5A05Al after removing corrosion products at different NSS corrosion durations: (**a-1**–**a-3**) 24 h; (**b-1**–**b-3**) 72 h; (**c-1**–**c-3**) 144 h; (**d-1**–**d-3**) 240 h; (**e-1**–**e-3**) 360 h.

SEM images in Figure 6 reveal the microstructure of the TAD_3D_/5A05Al interface after corrosion removal. To observe fine details, the interface is magnified. Figure 6a-1–a-3 depicts the microstructure after 24 h of NSS corrosion, showing pitting on the surface of the 5A05Al alloy, with pit sizes ranging from 2 μm to 8 μm, and minor corrosion. Figure 6b-1–b-3 displays the microstructure after 72 h of NSS corrosion. Compared to Figure 6a-1–a-3, the pits deepen, cracks with lengths of 10 μm to 20 μm appear inside the pits, cracks extend outward from the pits, and a small amount of aluminum oxide film peels off. Figure 6c-1–c-3 shows the microstructure after 144 h of NSS corrosion, revealing widespread pitting on the material surface, with fine cracks of lengths ranging from 20 μm to 80 μm on the pits. Some areas of the 5A05Al matrix are covered by corrosion products, while others are exposed again, indicating a decrease in corrosion resistance with extended NSS time. Figure 6d-1–d-3 reveals the microstructure after 240 h of NSS corrosion, showing a small number of pits on the metal surface covered by aluminum oxide. Compared to 144 h, some corrosion healing occurs in the passive film. Figure 6e-1–e-3 shows the microstructure after 360 h of NSS corrosion, where the 5A05Al matrix generates a large amount of MgAl_2_O_4_, Al(OH)_3_, and Al_2_O_3_. These aluminum oxides, along with the TAD_3D_ framework, form a dense corrosion product film, exhibiting a self-healing phenomenon in the composite material and enhancing corrosion resistance.

**Figure 7:**
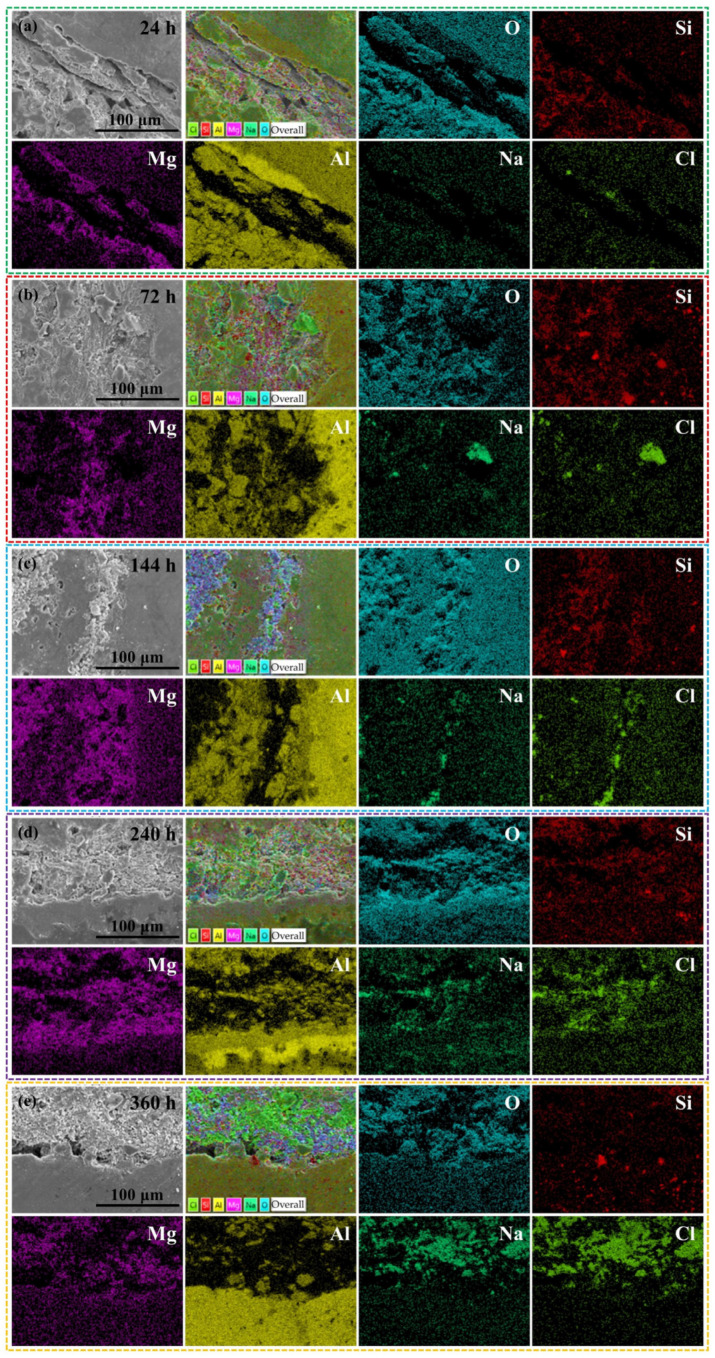
EDS of TAD_3D_/5A05Al after removing corrosion products at different NSS corrosion durations: (**a**) 24 h; (**b**) 72 h; (**c**) 144 h; (**d**) 240 h; (**e**) 360 h.

Figure 7 shows the EDS surface scan results of the TAD_3D_/5A05Al interface after corrosion removal, allowing the determination of the chemical composition of the corroded surface of the sample.

Figure 7a displays the EDS results after 24 h of NSS corrosion, indicating that the corrosion product is primarily composed of O, Si, Mg, Na, and Al elements. This corresponds to the corrosion-generated Al(OH)_3_ in the pits at the crack locations, causing minor damage to the passive film. The precipitated corrosion product is Al(OH)_3_ or corrosion-generated phases containing Al.

Figure 7b presents the EDS results after 72 h of NSS corrosion, indicating a higher content of Mg elements on the exposed matrix, suggesting the breakdown of the passive film. Compared to 5A05Al, the anodic oxidation of the β-phase Mg_5_Al_8_ leads to a higher sensitivity to the nucleation of early pitting micropores. Therefore, pits are formed in 5A05Al, and residual Na and Cl elements aggregate within these micropores.

Figure 7c presents the EDS results after 144 h of NSS corrosion, indicating that the surface product Al(OH)_3_ decomposes into aluminum oxides. Precipitated phases like Mg_2_Si and Si particles preferentially precipitate at grain boundaries, leading to the depletion of solute atoms in the 5A05Al matrix near the grain boundaries, forming precipitate-free zones (PFZs) on both sides along the grain boundaries. Since atoms like Si and Mn usually have higher potentials, they act as cathodes in the corrosion microcells. The precipitated phases exhibit a continuous distribution along the grain boundaries, forming a continuous cathode, while the PFZs formed near the precipitated phases are approximated as Al. Aluminum, with generally lower potential, acts as an anode in the corrosion microcell. Consequently, the PFZs can be rapidly dissolved in this corrosion microcell, resulting in corrosion [29]. The areas where the oxide film is damaged act as anodic sites in the active state, while those undamaged maintain a passive state as cathodic sites, forming an active–passive corrosion cell. The oxidation-reduction reactions cause metal dissolution within the pores to maintain internal electrical neutrality. Cl^−^ migrates into the pores, lowering the pH. Under the influence of H^+^ and Cl^−^, the metal is in an active state, leading to the formation of an active (inside the pore)–passive (outside the pore) corrosion cell. The increased migration of Cl^−^ and the decreased pH promote corrosion.

Figure 7d shows the EDS results after 240 h of NSS corrosion, indicating that the thin layer of interface reaction products formed at the interface of TAD_3D_ and 5A05.

Figure 7e shows the EDS results after 360 h of NSS corrosion, indicating that the corrosion products at the interface are primarily composed of O and Al. The corrosion products of the TAD_3D_/5A05Al composite material are mainly aluminum metal corrosion products. The corrosion products exhibit a point-like distribution, adsorbing at the interface between the TAD skeleton and the Al matrix, forming a dense surface oxide layer. The Al_2_O_3_ in the passive layer is largely an insulator for electron conduction, hindering localized corrosion and preventing the formation and expansion of corrosion-related defects such as pitting and cracks. The interpenetrating phase structure of IPC composite materials enhances the stability of the microstructure and improves overall corrosion resistance by forming a protective oxide layer.

Therefore, the NSS corrosion from 24 h to 360 h experienced processes of pitting corrosion and crevice corrosion. Self-healing of the passivation film became apparent at 240 h of corrosion, and at 360 h, the self-healing phenomenon was pronounced. This aligns with the conclusions drawn from SEM and EDS observations in Figure 4 and Figure 5.

### 3.4. Electrochemical Test Results of the Composite Material at Different NSS Corrosion Durations

**Figure 8:**
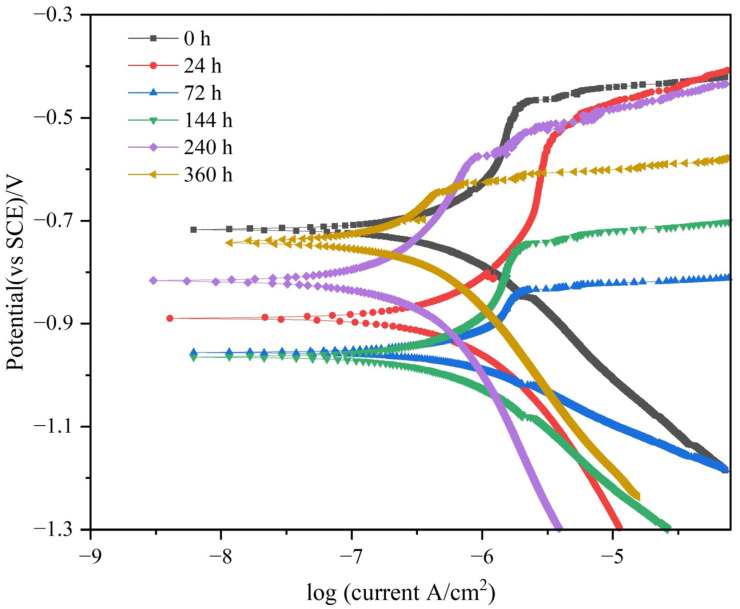
PDP of TAD_3D_/5A05Al with corrosion products after different NSS durations.

Figure 8 depicts the PDP curves of TAD_3D_/5A05Al at different times after NSS. It can be observed that the PDP trends of TAD_3D_/5A05Al are consistent at different times after NSS. The *E*_corr_ and *I*_corr_ values can be obtained from the PDP curve for Table 3. The *E*_corr_ value serves as a thermodynamic criterion for the corrosion resistance of the composite material; a higher *E*_corr_ value indicates stronger corrosion resistance, while a lower *E*_corr_ value suggests weaker corrosion resistance [30]. From Table 3, it can be seen that as the NSS corrosion time increases from 0 to 144 h, the *E*_corr_ value decreases. After 144 h of NSS corrosion, *E*_corr_ decreases to the minimum value of −0.971 V, indicating that the passive film is damaged, the corrosion voltage decreases, and the corrosion resistance reaches its lowest point. From 240 h to 360 h of NSS corrosion, the *E*_corr_ value increases. After 360 h of NSS corrosion, *E*_corr_ increases to −0.786 V, slightly lower than the *E*_corr_ value of the uncorroded sample (−0.718 V). This indicates that after 240 h of NSS, a large amount of corrosion products is formed on the surface of the composite material, protecting it. The corrosion voltage increases, the corrosion rate decreases, and the corrosion resistance improves. When NSS reaches 360 h, a complete corrosion product film forms on the surface. The corrosion voltage continues to increase, playing a suppressive role in corrosion, and enhancing corrosion resistance.

*I*_corr_ can serve as a dynamic criterion for the corrosion resistance of composite materials. A higher *I*_corr_ value indicates weaker corrosion resistance, while a lower *I*_corr_ value indicates stronger corrosion resistance. The trend of *I*_corr_ changes in Table 3 is also used to assess the corrosion resistance of the composite material, and the pattern is similar to that of *E*_corr_. As the NSS corrosion time increases from 0 to 144 h, the *I*_corr_ value increases. After 144 h of NSS corrosion, *I*_corr_ increases to the maximum value of 0.692 µA·cm^−2^, indicating that the passive film is damaged, the corrosion current is at its maximum, the corrosion rate increases, and the corrosion resistance reaches its lowest point. From 240 to 360 h of NSS corrosion, the *I*_corr_ value decreases. After 240 h of NSS corrosion, *I*_corr_ decreases to 0.178 µA·cm^−2^, which is already lower than the *I*_corr_ value of the uncorroded sample (0.398 µA·cm^−2^). After 360 h of NSS corrosion, *I*_corr_ decreases to 0.141 µA·cm^−2^, lower than the *I*_corr_ value of the uncorroded sample. After 240 h of NSS, the composite material’s surface begins to generate a large amount of corrosion products, providing protection. The corrosion current decreases, corrosion rate decreases, and corrosion resistance enhances. When NSS reaches 360 h, a complete corrosion product film forms on the surface, further reducing the corrosion current, suppressing corrosion, decreasing the corrosion rate, and improving corrosion resistance. Additionally, after NSS treatment, a dense oxide film forms on the surface of the TAD_3D_/5A05Al composite, increasing the anodic Tafel slope and decreasing the cathodic Tafel slope. This indicates that the presence of the oxide film alters the corrosion reaction kinetics of the aluminum alloy, reducing the rates of cathodic and anodic reactions.

**Figure 9:**
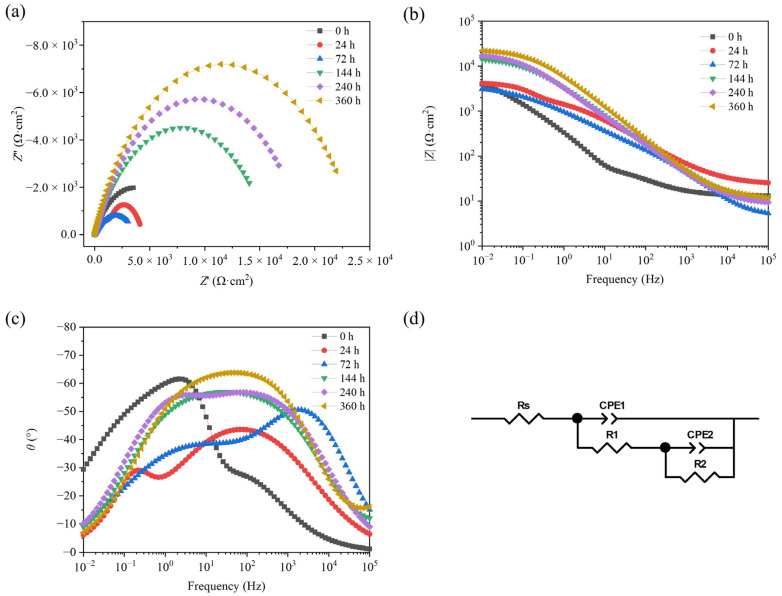
EIS of TAD_3D_/5A05Al with corrosion products after different NSS durations: (**a**) Nyquist diagram; (**b**) Bode diagram (|*Z*| − *F*); (**c**) Bode diagram (*θ* − *F*); (**d**) equivalent circuit.

Figure 9 shows the fitted results of EIS tests on TAD_3D_/5A05Al with corroded products after different NSS durations. Figure 9a shows the Nyquist plot of TAD_3D_/5A05Al, indicating a trend of decreasing and then increasing high-frequency arc radius with increasing NSS time. Since the arc radius is proportional to corrosion resistance, the corrosion resistance decreases and then increases with increasing NSS time. This is because the presence of O_2_ in the solution promotes the formation of the passive film on the surface of the 5A05Al alloy, but the film formation rate is slow in the early stages. Once a complete corrosion product film is formed, corrosion resistance begins to increase. Figure 9b,c depict the Bode plots of TAD_3D_/5A05Al. The impedance values of samples at different NSS times in the Bode plots exhibit a trend of initially decreasing and then increasing, consistent with the results from PDP. After NSS for 24 h, TAD_3D_/5A05Al is under the protection of a passive film, exhibiting excellent corrosion resistance. Between NSS 72 h and 144 h, with the increasing NSS time, the arc radius decreases, the peak value of the phase angle in the low-frequency region decreases, the height of the phase angle peak in the high-frequency region decreases, and its width narrows. Additionally, the slope of log *f* versus |*Z*| modulus decreases, and the impedance modulus value decreases [31]. All these indicate that the electrode is in the anodic dissolution stage, and the corrosion activity on the electrode surface becomes more intense. This suggests that the passive film of TAD_3D_/5A05Al is damaged, leading to a reduction in corrosion resistance. The sample corroded for 144 h. It is possible that, although the corrosion current is still high and the corrosion potential is still low, the passivation film of the composite material has begun to form; moreover, EIS shows an increase in the obstruction of the composite. After NSS for 240 h, there are more corrosion products on the 5A05Al alloy. Corrosion products such as MgAl_2_O_4_, Al(OH)_3_, Al_2_O_3_, and AlCl_3_ adsorb at the interface and, together with TAD_3D_ ceramics, form a protective oxide film, reducing the unevenness of the microelectrochemical environment and the pitting sensitivity on the surface of the composite material. The corrosion process of the composite material involves the inhibition of both anodic dissolution and cathodic depolarization reactions. Due to the abundant corrosion products and their rapid generation, approaching or exceeding the diffusion rate, the arc radius increases. The peak value of the phase angle in the low-frequency region increases, and its width widens. The slope of log *f* versus |*Z*| modulus increases, and the impedance modulus value increases. All these indicate that the corrosion resistance begins to enhance. By NSS 360 h, the low-frequency impedance modulus value increases, the arc radius begins to increase, and the peak phase angle region widens. This indicates a steady improvement in the corrosion resistance of TAD_3D_/5A05Al, as the surface corrosion products are most abundant and dense, and the surface passivation film is fully formed, achieving optimal corrosion resistance. Figure 9d illustrates the equivalent circuit diagram used during the fitting process. By analyzing the EIS spectra, it can be concluded that all the curves exhibited two capacitive loops. It can be assumed that overall response of the system is associated with (*R*_1_–*CPE*_1_) loop owing to the formation of film on the alloy surface and (*R*_2_–*CPE*_2_) loop due to the double layer formed at metal–solution interface. Among them, Rs is the resistance of the electrolyte, *CPE*_1_ is the oxide film capacitance, *R*_1_ is the oxide film resistance, *CPE*_2_ is the double-layer capacitance, and *R*_2_ is the charge transfer resistance. The 1st loop (*R*_1_–*CPE*_1_) encompasses all the information related to the surface layer and the possible defects that may be present within it. The EIS results were analyzed with the ZView program using the equivalent circuit shown in Figure 9d, and the values of the parameters obtained are tabulated in Table 4.

From Table 4, it can be observed that the solution resistance (*R*_s_) remained constant. The results showed that the increases in impedance values were strongly linked by the values of *R*_1_ and *R*_2_. Further, a slight deviation from typical capacitive behavior at high frequencies, possibly due to uneven oxide film formation on the metal matrix. With different times of NSS corrosion treatment, the *R*_2_ value of the TAD_3D_/5A05Al increases. Without NSS, the *R*_2_ value of the sample is the lowest, at 7.30 × 10^3^ Ω·cm^2^. After NSS 360 h, the *R*_2_ value of the sample is the highest, at 2.40 × 10^4^ Ω·cm^2^. *R*_1_ and *R*_2_ play a decisive role in the impedance value of the sample, and compared to the values of *R*_1_ and *R*_2_, the *R*_s_ value can be basically ignored.

It is worth noting that the presence of the second phase/interface phase typically disrupts the continuity of the passivation film, and the vicinity of the second phase/interface phase often becomes a weak point in the passivation film. The presence of corrosive ions, especially Cl ions, can lead to the rupture of the passivation film, thereby triggering galvanic corrosion/pitting initiation. Specifically, the presence of Mg in 5A05Al can reduce the surface tension of the Al liquid and the surface energy between the Al liquid and TAD_3D_, improving the wetting properties between them. This enables the preparation of composite materials with lower porosity and higher density, indirectly suppressing the direct interface reaction between TAD_3D_ and Al liquid and the formation of harmful interface reaction phases. Meanwhile, electrochemical tests indicate that factors such as lower interface corrosion resistance or a relatively weak passivation film at the interface do not significantly reduce the corrosion resistance of TAD_3D_ and 5A05Al. This is because the TAD_3D_/5A05Al composite material has a unique IPC structure. The Mg in 5A05Al improves the wetting properties between the Al liquid and TAD_3D_ and simultaneously forms a macroscopic interface composite with TAD_3D_. When immersed in a corrosive medium containing ions, before the formation of a chemical oxidation film, oxygen and electrons freely diffuse and migrate at the interface between the solution and the metal. Therefore, the anodic and cathodic corrosion rates of TAD_3D_/5A05Al are relatively high. After the formation of the chemical oxidation film, this uniformly dense film hinders the diffusion and migration of O_2_ and electrons between the solution and the metal interface, suppressing both anodic and cathodic reactions. As a result, the corrosion resistance of the aluminum alloy is improved, ensuring that the overall corrosion resistance of the composite material is not reduced. This is consistent with the microscopic morphology of the oxide film observed by SEM and EDS chemical composition analysis.

## 4. Conclusions

In this investigation, the corrosion behavior of TAD_3D_/5A05Al composite material in a chloride environment was investigated through various methods, including SEM, EDS, and electrochemical tests. The conclusions are as follows:

1. Processed aluminum dross is used as the raw material, and the sintered TAD_3D_ framework has a pore size of about 10 ppi. During the pressureless melt infiltration process, the TAD_3D_ framework guides the molten 5A05Al alloy, effectively expelling fine air bubbles between the framework and the 5A05Al solution, preventing defects inside the material. The interface between TAD_3D_ and 5A05Al is tightly bonded, exhibiting strong interface adhesion.
2. SEM and EDS indicate that during the NSS corrosion from 24 h to 360 h, the composite material undergoes pitting corrosion, crevice corrosion and self-healing processes. Self-healing of the passivation film became apparent at 240 h of corrosion, and at 360 h, the self-healing phenomenon was pronounced. This is attributed to the unique IPC structure of the TAD_3D_/5A05Al composite. The macroscopic interface between 5A05Al and TAD_3D_ facilitates the formation of corrosion products such as MgAl_2_O_4_, Al(OH)_3_, Al_2_O_3_, and AlCl_3_ adsorbed at the interface. Together with TAD_3D_ ceramics, they form an oxide film, reducing the unevenness of the microelectrochemical environment and sensitivity to pitting.
3. PDP tests indicate that the corrosion current density of TAD_3D_/5A05Al increases and then decreases after NSS 0 h, 24 h, 72 h, 144 h, 240 h, and 360 h, suggesting that the corrosion resistance initially decreases and then increases. *E*_corr_ decreases from −0.718 V to −0.786 V, and *I*_corr_ decreases from 0.398 μA·cm^−2^ to 0.141 μA·cm^−2^. After NSS treatment, a dense oxide film forms on the surface of the TAD_3D_/5A05Al composite, enhancing the anodic Tafel slope while reducing the cathodic Tafel slope. This indicates that the presence of the oxide film alters the corrosion reaction kinetics of the aluminum alloy, reducing the rates of cathodic and anodic reactions.
4. EIS test results indicate that with an increase in NSS time, the high-frequency arc radius shows a trend of initially decreasing and then increasing. The impedance values in the Bode plot also exhibit a trend of initially decreasing and then increasing. This suggests that factors such as lower interface corrosion resistance or a relatively weak passivation film at the interface do not significantly reduce the corrosion resistance of TAD_3D_ and 5A05Al. Instead, the corrosion resistance of the composite material decreases initially and then increases.

The study has demonstrated the potential use of TAD_3D_/5A05Al in a chloride environment. In future work, it is advisable to extend the duration of NSS corrosion testing to observe whether the material’s corrosion resistance can be further enhanced. Attempts to protect the surface of composite materials using methods such as micro-arc oxidation. Additionally, further investigation into the passivation self-healing mechanism and corrosion resistance mechanisms of the composite material is warranted.

## Figures and Tables

**Table 1:** Composition of treated aluminum dross (TAD) and kaolin (mass fraction).

Composition	Al_2_O_3_	SiO_2_	MgO	CaO	TiO_2_
TAD	87.54	3.06	5.15	1.08	0.15
kaolin	42.23	56.52	0.55	0.49	0.21

**Table 2:** Composition of 5A05 Al alloy (mass fraction).

Elements	Mg	Si	Zn	Mn	Cu	Fe	Al
wt.%	5.02	0.10	0.04	0.43	0.05	0.21	Balance

**Table 3:** Corrosion potential (*E*_corr_) and corrosion current density (*I*_corr_) of TAD_3D_/5A05Al under different NSS corrosion durations.

NSS Corrosion Durations	*E*_corr_ (V vs. SCE)	*I*_corr_ (µA·cm^−2^)
0 h	−0.718	0.398
24 h	−0.885	0.537
72 h	−0.957	0.646
144 h	−0.971	0.692
240 h	−0.839	0.178
360 h	−0.786	0.141

**Table 4:** Electrochemical impedance parameters of TAD_3D_/5A05Al under different NSS corrosion durations.

NSS Corrosion Durations	*R*_s_ (Ω·cm^2^)	*CPE*_1_ (F·cm^−2^)	*n*_1_	*R*_1_ (Ω·cm^2^)	*CPE*_2_ (F·cm^−2^)	*n*_2_	*R*_2_ (Ω·cm^2^)
0 h	12.96	7.96 × 10^−4^	0.63	7.84 × 10^1^	8.57 × 10^−5^	1.13	7.30 × 10^3^
24 h	23.07	1.25 × 10^−4^	0.58	2.36 × 10^3^	5.22 × 10^−4^	1.05	2.06 × 10^3^
72 h	4.56	3.08 × 10^−5^	0.75	1.28 × 10^2^	3.35 × 10^−4^	0.50	3.67 × 10^3^
144 h	9.62	1.40 × 10^−9^	1.34	2.17 × 10^0^	7.81 × 10^−5^	0.66	1.58 × 10^4^
240 h	8.41	7.21 × 10^−5^	0.68	4.69 × 10^3^	9.87 × 10^−6^	0.91	1.43 × 10^4^
360 h	8.73	1.51 × 10^−5^	0.55	1.46 × 10^1^	2.67 × 10^−5^	0.76	2.40 × 10^4^

## Data Availability

Data are contained within the article
